# A multicenter, double-blind, randomized, controlled phase III clinical trial of chicken type II collagen in rheumatoid arthritis

**DOI:** 10.1186/ar2870

**Published:** 2009-12-01

**Authors:** Wei Wei, Ling-Ling Zhang, Jian-Hua Xu, Feng Xiao, Chun-De Bao, Li-Qing Ni, Xing-Fu Li, Yu-Qing Wu, Ling-Yun Sun, Rong-Hua Zhang, Bao-Liang Sun, Sheng-Qian Xu, Shang Liu, Wei Zhang, Jie Shen, Hua-Xiang Liu, Ren-Cheng Wang

**Affiliations:** 1 Institute of Clinical Pharmacology, Anhui Medical University, Key Laboratory of Anti-inflammatory and Immunopharmacology of Education Ministry, 81 Meishan Road, Hefei 230032, PR China; 2 Rheumatism and Immunity Department, The First Affiliated Hospital of Anhui Medical University, 218 Jixi Road, Hefei 230022, PR China; 3 Rheumatism and Immunity Department, The Affiliated Shanghai Renji Hospital of Shanghai Jiao Tong University, 1630 Dongfang Road, Shanghai 200127, PR China; 4 Rheumatism and Immunity Department, Shanghai Guanghua Hospital, 540 Xinhua Road, Shanghai 200052, PR China; 5 Rheumatism and Immunity Department, Qilu Hospital of Shandong University, 107 Wenhua Road, Jinan 250012, PR China; 6 Rheumatism and Immunity Department, The Third Affiliated Hospital of Sun Yat-Sen University, 600 Tianhe Road, Guangzhou 510630, PR China; 7 Rheumatism and Immunity Department, The Affiliated Drum Tower Hospital of Nanjing University Medical School, 321 Zhongshan Road, Nanjing 210008, PR China; 8 Rheumatism and Immunity Department, Southwest Hospital of Third Military Surgeon University, 30 Shapingba Gaotanyan Street, Chongqing 400038, PR China; 9 Rheumatism and Immunity Department, The Affiliated Hospital of Taishan Medical College, 706 Tanshan Street, Taian 271000, PR China

## Abstract

**Introduction:**

Chicken type II collagen (CCII) is a protein extracted from the cartilage of chicken breast and exhibits intriguing possibilities for the treatment of autoimmune diseases by inducing oral tolerance. A 24-week, double-blind, double-dummy, randomized, methotrexate (MTX)-controlled study was conducted to evaluate the efficacy and safety of CCII in the treatment of rheumatoid arthritis (RA).

**Methods:**

Five hundred three RA patients were included in the study. Patients received either 0.1 mg daily of CCII (n = 326) or 10 mg once a week of MTX (n = 177) for 24 weeks. Each patient was evaluated for pain, morning stiffness, tender joint count, swollen joint count, health assessment questionnaire (HAQ), assessments by investigator and patient, erythrocyte sedimentation rate (ESR), and C-reactive protein (CRP) by using the standard tools at baseline (week 0) and at weeks 12 and 24. Additionally, rheumatoid factor (RF) was evaluated at weeks 0 and 24. Measurement of a battery of biochemical parameters in serum, hematological parameters, and urine analysis was performed to evaluate the safety of CCII.

**Results:**

Four hundred fifty-four patients (94.43%) completed the 24-week follow-up. In both groups, there were decreases in pain, morning stiffness, tender joint count, swollen joint count, HAQ, and assessments by investigator and patient, and all differences were statistically significant. In the MTX group, ESR and CRP decreased. RF did not change in either group. At 24 weeks, 41.55% of patients in the CCII group and 57.86% in the MTX group met the American College of Rheumatology 20% improvement criteria (ACR-20) and 16.89% and 30.82%, respectively, met the ACR 50% improvement criteria (ACR-50). Both response rates for ACR-20 and ACR-50 in the CCII group were lower than those of the MTX group, and this difference was statistically significant (*P *< 0.05). The DAS28 (disease activity score using 28 joint counts) values of the two treatment groups were calculated, and there was a statistically significant difference between the two treatment groups (*P *< 0.05). Gastrointestinal complaints were common in both groups, but there were fewer and milder side effects in the CCII group than in the MTX group. The incidence of adverse events between the two groups was statistically significant (*P *< 0.05).

**Conclusions:**

CCII is effective in the treatment of RA and is safe for human consumption. CCII exerts its beneficial effects by controlling inflammatory responses through inducing oral tolerance in RA patients.

**Trials Registration:**

Clinical trial registration number: ChiCTR-TRC-00000093.

## Introduction

Rheumatoid arthritis (RA) is a chronic inflammatory disease characterized by pain, swelling, and stiffness of multiple joints. It is also a highly disabling disease that limits mobility, hampers work, and reduces quality of life. Chronic inflammation commonly results in progressive joint destruction, deformity, and loss of function. Complex immune mechanisms contribute to the pathology of RA [1,2]. Current pharmacological strategies addressing mainly immune suppression and anti-inflammatory mechanisms have had limited success. Currently, most drugs for RA are steroids, non-steroidal anti-inflammatory drugs (NSAIDs), disease-modifying drugs, and biological agents. These therapies are associated with significant side effects with long administration, including anorexia, dyspepsy, suppression of the immune system non-specifically, and infections [3-5].

Recently, more and more oral tolerance mechanisms have been studied in the treatment of autoimmune diseases. Oral tolerance has posed intriguing possibilities for the treatment of autoimmune diseases, including RA. Oral tolerance is a state of systemic immune suppression to an antigen induced by oral feeding of the same antigen. Extensive research in this area over the past 10 years has led to the conclusion that two mechanisms are operative in the mediation of oral tolerance: active suppression and clonal anergy or deletion. A number of factors that determine which mechanisms of tolerance are operative have been identified: antigen dose, antigen form, and the timing of antigen administration [6,7].

Oral administration of autoantigen has been shown to suppress a variety of autoimmune pathologies induced experimentally, including antigen-induced RA [8]. Modulating the immune response to the autoantigen by oral tolerance may be a safer and more effective treatment. A number of candidate autoantigens have been identified in RA [9]. Type II collagen (CII) is a major protein in articular cartilage and a potential autoantigen. Some RA patients demonstrate immunity against CII, and autoantibodies to CII have been detected in the sera of both pauciarticular-onset and systemic-onset RA patients [10]. These data support the view that autoimmunity to an antigen such as CII in cartilage plays a major role in the pathogenesis of RA. In animal models, oral administration of CII prevents and reduces the severity of autoimmune diseases [11]. Work from these animal models has recently been extended into human clinical trials of RA with differing degrees of success [12-14]. Hence, oral tolerance has been advocated as a treatment strategy for autoimmune diseases, including RA.

Investigators in our laboratory found that collagen-induced arthritis (CIA) could be established in Wistar rats, Kunming mice, and DBA/1 mice with chicken type II collagen (CCII) [15,16]. Feeding CCII to rats by oral administration decreased the arthritis index. Meanwhile, cartilage degeneration, synovium hyperplasia, and inflammatory cell infiltration in the knee joints of mice and rats with CIA were suppressed by CCII [17,18]. These experiments in rodents have provided the basis for human clinical trials. In a randomized, double-blind, multicenter, and controlled phase II clinical trial involving 236 patients with severe active RA, a decrease in the number of swollen joints and tender joints occurred in subjects fed CCII for 6 months. Meanwhile, CCII could reduce pain, morning stiffness, health assessment questionnaire (HAQ), and assessments by investigator and patient, and the incidence of adverse events of CCII was lower than that of methotrexate (MTX) [19]. These results demonstrate clinical efficacy of an oral tolerance approach for RA. To evaluate the efficacy and safety of CCII in RA patients further, we treated two groups of RA patients with oral CCII or MTX in a randomized, double-blind, multicenter, and controlled phase III clinical trial.

## Materials and methods

### Recruitment of patients

This trial was performed at eight centers from October 2004 to December 2005 (clinical trial registration number: ChiCTR-TRC-00000093). The study protocol was evaluated and approved by their respective investigational and ethics committees. Five hundred three intention-to-treat (ITT) population RA patients (18 to 65 years old) who met revised American College of Rheumatology (ACR) criteria for the diagnosis of RA were entered into the study after giving their written informed consent [20]. There are no patients in the phase II study who were enrolled in this phase III trial. Table 1 defines the study population. Admission criteria also included patients of either gender with RA with a duration of 6 to 24 months. Active RA was defined as the presence of at least three of the following criteria: six or more painful or tender joints, three or more swollen joints, morning stiffness for at least 45 minutes (on average during the week prior to entry), and an erythrocyte sedimentation rate (ESR) of at least 28 mm. Second-line agents were discontinued at least 4 weeks prior to entry. Continuous doses of NSAIDs were permitted. Patients to whom one of following applied were excluded: dysfunction of liver; severe cardiovascular, urinary, hematopoietic, or endocrine system disease; immunodeficiency; uncontrolled infection or active gastrointestinal tract disease; recent vaccination; gravida; women in lactation period or those recently intending to become pregnant; hypersensitivity to CII; treatment with any other disease-modifying anti-rheumatic drug within 30 days before enrolment; history of alcohol abuse; history of hyperglycemia or motor coordination disorder; or participation in other clinical trials within 3 months before enrolment.

**Table 1:** Comparison of baseline clinical characteristics between the chicken type II collagen group and the methotrexate group

Variables	CCII (n = 296)	MTX (n = 158)	*P *value
Gender, male/female	55/241	30/128	0.983
Age, years	47.11 ± 10.55	47.06 ± 11.14	0.967
Duration of rheumatoid arthritis, years	1.63 ± 0.75	1.72 ± 0.52	0.949
Body temperature, °C	36.66 ± 0.38	36.65 ± 0.39	0.892
Pain (VAS^a^)	6.02 ± 1.43	5.91 ± 1.76	0.528
Morning stiffness, minutes	99.26 ± 25.14	104.89 ± 26.42	0.277
Tender joint count	13.34 ± 6.43	14.09 ± 6.82	0.233
Swollen joint count	10.38 ± 6.63	10.57 ± 7.25	0.773
HAQ^b^	0.82 ± 0.56	0.86 ± 0.55	0.454
Physician's assessment (VAS)	5.83 ± 1.54	5.78 ± 1.26	0.892
Patient's assessment (VAS)	6.01 ± 1.51	6.06 ± 1.71	0.731
ESR^c^, mm/hour	38.18 ± 27.58	42.51 ± 29.37	0.118
C-reactive protein, mg/L	17.52 ± 8.27	23.24 ± 9.81	0.306
Rheumatoid factor, U/mL	203.65 ± 61.95	198.99 ± 21.31	0.115

The same variables were compared between the chicken type II collagen (CCII) group and the methotrexate (MTX) group. Fisher exact test was used for categorical variables, and *t *analysis of variance was used for continuous variables. ^c^ESR, erythrocyte sedimentation rate; ^b^HAQ, health assessment questionnaire; MTX, methotrexate; ^a^VAS, visual analogue scale.

### Study design

The study was a two-to-one, eight-center, 24-week follow-up, double-blind, double-dummy, randomized, and MTX-controlled trial comparing efficacy and safety of CCII and MTX in the treatment of RA. Patients were randomly assigned to a CCII (n = 326) or MTX (n = 177) group that received either CCII (0.1 mg daily) or MTX (10 mg once a week). Patients and investigators were blinded to the treatment regimens throughout the study. Efficacy variables were assessed at 0, 12, and 24 weeks after administration of drug. Patients were allowed to remain on diclofenac sodium (50 mg daily), an NSAID. The diclofenac sodium dosage was not changed during the study. CCII capsules (#040328; Shanghai Materia Medica Bioengineering Institute, Shanghai, China), CCII dummy capsules, MTX tablets (#031201; Shanghai Xin Yi Pharmaceutical Factory, Shanghai, China), and MTX dummy tablets were obtained from Shanghai Materia Medica Bioengineering Institute. Patients were instructed to take oral CCII capsules or dummy capsules with 200 mL of cold water 30 minutes prior to eating breakfast every morning.

### Flow sheet of production of chicken type II collagen

**Figure 1:**
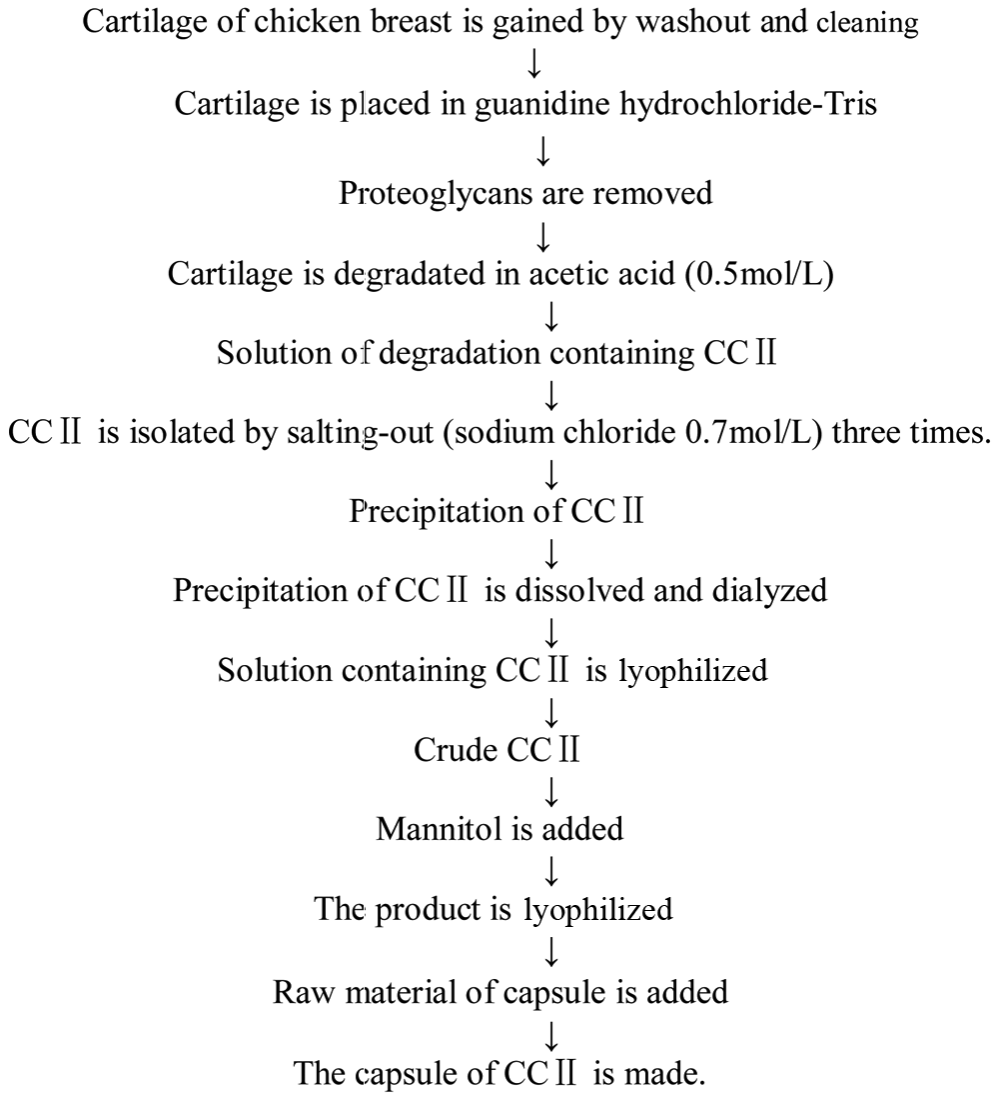
The flow sheet of production of chicken type II collagen (CCII). CCII is a protein extracted from the cartilage of chicken breast. Its molecular weight is 115 to approximately 135 kDa by SDS-PAGE electrophoresis method. In this study, the CCII capsule that patients received consisted of CCII and an adjuvant such as mannitol and glidantin.

CCII is a protein extracted from the cartilage of chicken breast. Its molecular weight is 115 to approximately 135 kDa by SDS-PAGE electrophoresis method. In this study, the CCII capsule that patients received consisted of CCII and an adjuvant such as mannitol and glidantin. Figure 1 shows the flow sheet of production of CCII.

### Clinical assessments

Clinical assessments of efficacy were made at baseline and repeated 12 and 24 weeks later. Efficacy variables included [21,22] pain, and pain intensity was assessed by visual analogue scale (VAS) of 0 (no pain) to 10 (severe pain). Patients were questioned about the duration of morning stiffness experienced on the day before each study visit. Joint counts for tenderness and swelling were the sum of the number of affected joints. Physician and patient global assessments were rated according to VAS of 0 (very good) to 10 (very poor). Functional status was assessed at baseline and at 12 and 24 weeks using HAQ. ESR and C-reactive protein (CRP) values were obtained at baseline and at 12 and 24 weeks. Rheumatoid factor (RF) positivity was determined at the screening visit and at 24 weeks.

The primary efficacy variable was the ACR preliminary definition of improvement in RA [23]. To reach improvement according to the ACR definition, a patient with RA must improve by at least 20% in tender and swollen joint count and by at least 20% in three of the five other measures: patient global assessment, physician global assessment, HAQ, acute-phase reactant, and patient pain assessment. In addition to the evaluation of 20% improvement (ACR-20), we determined RA improvement based on more substantial changes in RA core set measures, such as requiring at least 50% improvement (ACR-50) reported as secondary efficacy measures. The disease activity score using 28 joint counts (DAS28) was evaluated [24]. Clinical parameters also included body weight, blood pressure, and heart rate. To standardize the evaluation of clinical variables, all investigators prior to study entry performed clinical evaluation of one patient with active RA.

### Adverse events

At each visit, the patient was asked whether side effects were noticed during the interim. Side effects such as gastrointestinal complaints, vomiting, anorexia, headache, dizziness, insomnia, tetter, and mouth ulcers were known to occur frequently in treatment with CCII or MTX. Moreover, at entry and at 12 and 24 weeks, the following laboratory variables were assessed to monitor safety: complete blood cell count, serum levels of liver enzymes, creatinine, uric acid, and urinalysis.

### Statistical analysis

**Figure 2:**
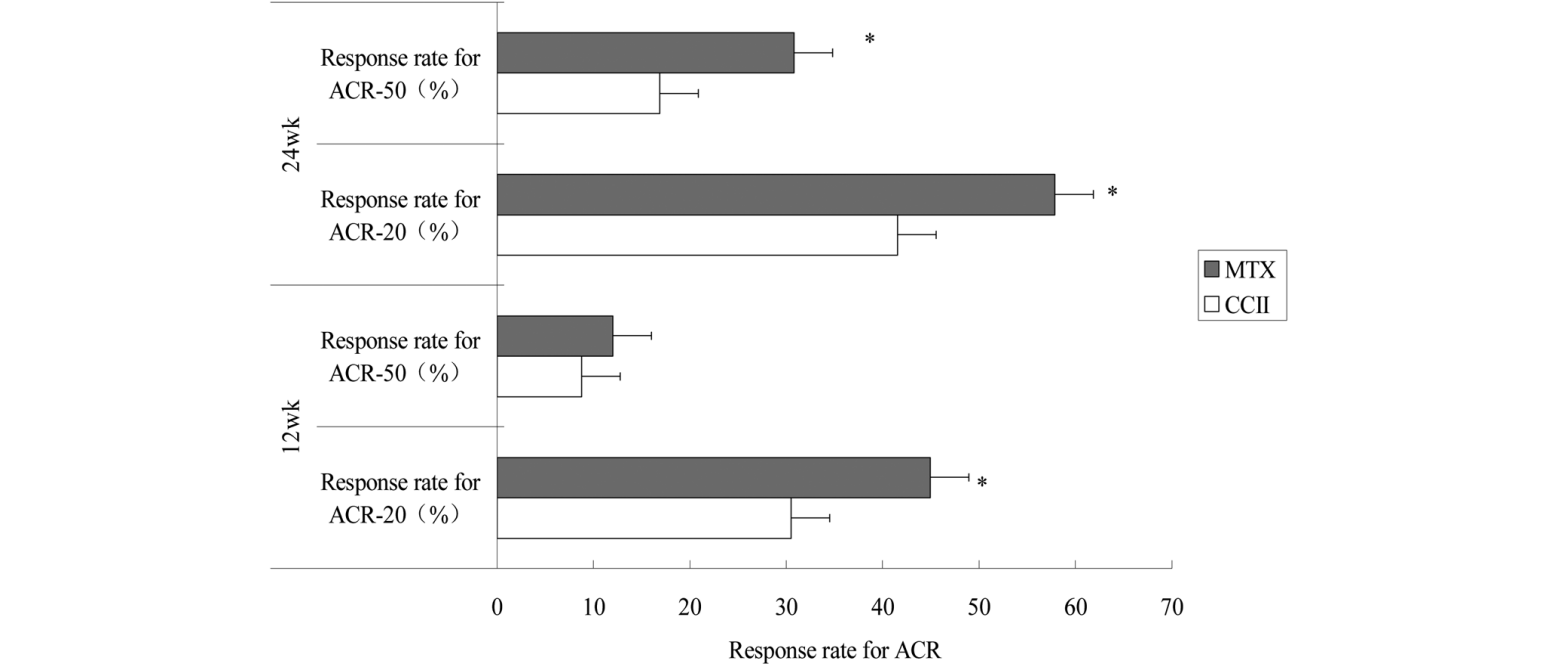
Comparison of the effect of American College of Rheumatology 20% improvement criteria (ACR-20) and ACR 50% improvement criteria (ACR-50) between two groups at 12 and 24 weeks. Response rates for ACR-20 and ACR-50 were assessed at 12 and 24 weeks. With an intention-to-treat analysis, response rates for ACR-20 were 30.51% in the chicken type II collagen (CCII) group and 44.94% in the methotrexate (MTX) group at 12 weeks. Response rates for ACR-50 were 8.81% and 15.03%, respectively. Response rates for ACR-20 were 41.55% in the CCII group and 57.86% in the MTX group at 24 weeks. Response rates for ACR-50 were 16.89% and 30.82%, respectively. Both response rates for ACR-20 and ACR-50 of the CCII group were lower than those of the MTX group. These changes were different to a statistically significant degree between the two treatment groups. *P < 0.05 versus CCII group.

Safety assessments were performed on all patients who consumed any masked study medication. Efficacy analyses were performed on the ITT population as well as on the population of patients who completed the 24-week study. Efficacy analysis of outcome variables was based on mean changes from baseline to endpoint in the ITT population. The data in Tables 1 and 2 and Figure 2 are expressed as mean ± standard deviation. The statistical software product used for these analyses was SAS, version 8.1 (SAS Institute Inc., Cary, NC, USA). All laboratory variables were subjected to descriptive statistics and compared by means of the Wilcoxon signed rank test. The randomization code was exposed only after the database was locked. Chi-square with Fisher exact test was used for categorical variables, and *t *analysis of variance was used for continuous variables. Significance level was established at 0.05.

**Table 2:** Results in outcome variables at entry and at 12 and 24 weeks

Outcome variables	CCII	*P *value^a^	MTX	*P *value^a^	*P *value^b^
Pain (VAS)					
Entry	6.02 ± 1.43		5.91 ± 1.76		>0.05
12 weeks	4.59 ± 2.22	<0.01	4.09 ± 1.99	<0.01	>0.05
24 weeks	3.58 ± 2.55	<0.01	3.38 ± 2.35	<0.01	<0.05
Morning stiffness, minutes					
Entry	99.26 ± 25.14		104.89 ± 26.42		>0.05
12 weeks	62.66 ± 25.06	<0.01	45.83 ± 21.28	<0.01	<0.01
24 weeks	36.12 ± 17.21	<0.01	33.98 ± 12.59	<0.01	>0.05
Tender joint count					
Entry	13.34 ± 6.43		14.09 ± 6.82		>0.05
12 weeks	9.14 ± 6.67	<0.01	8.78 ± 6.01	<0.01	>0.05
24 weeks	6.34 ± 4.81	<0.01	7.22 ± 6.91	<0.01	>0.05
Swollen joint count					
Entry	10.38 ± 6.63		10.57 ± 7.25		>0.05
12 weeks	6.89 ± 5.46	<0.01	5.63 ± 4.97	<0.01	<0.01
24 weeks	4.26 ± 2.03	<0.01	4.38 ± 2.94	<0.01	>0.05
HAQ					
Entry	0.82 ± 0.56		0.86 ± 0.55		>0.05
12 weeks	0.65 ± 0.41	<0.01	0.51 ± 0.42	<0.01	<0.01
24 weeks	0.43 ± 0.27	<0.01	0.44 ± 0.21	<0.01	<0.05
Physician's assessment (VAS)					
Entry	5.83 ± 1.54		5.78 ± 1.26		>0.05
12 weeks	4.68 ± 2.09	<0.01	4.03 ± 2.06	<0.01	<0.01
24 weeks	3.81 ± 1.52	<0.01	3.53 ± 1.64	<0.01	>0.05
Patient's assessment (VAS)					
Entry	6.01 ± 1.51		6.06 ± 1.71		>0.05
12 weeks	4.86 ± 2.01	<0.01	4.31 ± 2.06	<0.01	<0.01
24 weeks	3.92 ± 2.45	<0.01	3.71 ± 2.04	<0.01	<0.05
ESR^c^, mm/hour					
Entry	38.18 ± 17.58		42.51 ± 19.37		>0.05
12 weeks	38.03 ± 14.17	>0.05	35.84 ± 13.24	<0.01	<0.01
24 weeks	37.53 ± 10.22	>0.05	34.21 ± 15.25	<0.01	<0.01
C-reactive protein, mg/L					
Entry	17.52 ± 8.27		23.24 ± 9.81		>0.05
12 weeks	17.27 ± 15.14	>0.05	16.01 ± 13.41	<0.01	<0.05
24 weeks	15.56 ± 12.38	>0.05	15.08 ± 12.25	<0.01	<0.05
Rheumatoid factor, U/mL					
Entry	203.65 ± 61.95		198.99 ± 21.31		>0.05
24 weeks	150.21 ± 16.18	>0.05	123.35 ± 11.32	>0.05	>0.05

In both groups, there were decreases in pain, morning stiffness, tender joint count, swollen joint count, health assessment questionnaire (HAQ), and global assessment of efficacy by investigator and patient. Within-group differences (study entry versus 12 and 24 weeks) were statistically significant. In the methotrexate (MTX) group, erythrocyte sedimentation rate (ESR) and C-reactive protein decreased, but changes in the two variables in the chicken type II collagen (CCII) group were not significant. Rheumatoid factor was not significantly affected by either drug therapy. VAS, visual analogue scale. *P *value^a^, entry versus 12 or 24 weeks in CCII group or MTX group; *P *value^b^, CCII group versus MTX group;^c^ESR, erythrocyte sedimentation rate.

## Results

### Baseline characteristics

Of 503 randomly assigned patients (326 in the CCII group and 177 in the MTX group), 49 patients withdrew early. Thirty (9.20%) patients withdrew in the CCII group, and 19 (10.73%) patients withdrew in the MTX group. There were various reasons for early withdrawal, such as adverse events, non-compliance, lack of response, and loss at follow-up. Three patients (0.92%) in the CCII group reported side effects, and five patients (2.82%) in the MTX group reported side effects. In the CCII group, three patients (0.92%) withdrew due to lack of compliance, and four patients (2.25%) withdrew because of non-compliance in the MTX group. Twelve (3.68%) and four (2.25%) patients in the CCII group and in the MTX group, respectively, withdrew because of lack of efficacy. Twelve and six patients in the CCII group and in the MTX group, respectively, were lost to follow-up. Four hundred fifty-four patients (296 in the CCII group and 158 in the MTX group) completed 24 weeks of therapy. There were no statistically significant differences between the two groups in terms of adverse events, non-compliance, lack of response, and loss at follow-up. At study entry, the two groups were well balanced with regard to demographic characteristics and disease parameters, and there were no statistically significant differences between the two groups in terms of gender, age, disease duration, body temperature, pain, morning stiffness, tender joint count, swollen joint count, HAQ, physician's assessment, patient's assessment, ESR, CRP, and RF (Table 1). Also, there were no important differences among the eight centers.

### Efficacy

In both groups, there were decreases in pain, morning stiffness, tender joint count, swollen joint count, HAQ, and assessments of efficacy by both investigator and patient. Within-group differences (study entry versus 12 and 24 weeks) were statistically significant for the above clinical disease parameters (Table 2). At 12 weeks, there were statistically significant differences in morning stiffness, swollen joint count, HAQ, physician's assessment, and patient's assessment between the CCII group and the MTX group; there were statistically significant differences in pain, HAQ, and patient's assessment at 24 weeks between the two groups. In the MTX group, ESR and CRP decreased at 12 and 24 weeks, but changes in the two variables in the CCII group were not significant, and there was a statistically significant difference between the two groups. RF was not significantly affected by either drug therapy (Table 2).

### American College of Rheumatology response criteria

Response rates for ACR-20 and ACR-50 were assessed at 12 and 24 weeks. With an ITT analysis (Figure 2), response rates for ACR-20 were 30.51% in the CCII group and 44.94% in the MTX group at 12 weeks. Response rates for ACR-50 were 8.81% and 15.03%, respectively. Response rates for ACR-20 were 41.55% in the CCII group and 57.86% in the MTX group at 24 weeks. Response rates for ACR-50 were 16.89% and 30.82%, respectively. Both response rates for ACR-20 and ACR-50 of the CCII group were lower than those of the MTX group. These changes were different to a statistically significant degree between the two treatment groups (*P *< 0.05).

### Disease activity score using 28 joint counts

The DAS28 values of the two treatment groups were calculated (Table 3), and the results showed that the numbers of relief patients (≤ 2.6), low-activity patients (2.6 to approximately 3.2), mid-activity patients (3.2 to approximately 5.1), and high-activity patients (>5.1) were 36, 26, 138, and 96, respectively, in the CCII group at 24 weeks. In the MTX group, the numbers of relief patients, low-activity patients, mid-activity patients, and high-activity patients were 20, 19, 88, and 31, respectively. There was a statistically significant difference between the two treatment groups (*P *< 0.05).

**Table 3:** Comparison of DAS28 between the chicken type II collagen group and the methotrexate group

Group	Relief (≤ 2.6)	Low activity (2.6~3.2)	Mid activity (3.2~5.1)	High activity (>5.1)	Chi-square	*P *value^a^
CCII (n = 296)	36	26	138	96	5.476	0.019
MTX (n = 158)	20	19	88	31		

^a^Showed rank sum test. CCII, chicken type II collagen; DAS28, disease activity score using 28 joint counts; MTX, methotrexate.

### Adverse events

All trials reported data about drug-related adverse outcomes in the ITT population. The majority of adverse outcomes were mild to moderate disturbances of the gastrointestinal tract. Gastrointestinal complaints were the most common adverse events. Other adverse events included vomiting, anorexia, headache, dizziness, insomnia, tetter, and mouth ulcers. These adverse events were mild and did not interfere with the continuation of treatment drugs (Table 4).

**Table 4:** Comparison of adverse events between the chicken type II collagen group and the methotrexate group at 12 and 24 weeks

Weeks	Groups	Adverse events	Rate of adverse events	Chi-square	*P *value^a^
12	CCII (n = 326)	18	5.52%	1.974	0.048
	MTX (n = 177)	15	8.47%		
24	CCII (n = 326)	18	5.52%	2.001	0.036
	MTX (n = 177)	17	9.60%		

^a^Showed chi-square test. CCII, chicken type II collagen; MTX, methotrexate.

The analysis of adverse events was carried out on all 503 randomly assigned patients, and the adverse events of all patients were generally well tolerated. Adverse events were common in both groups. During the treatment period, the CCII group reported 18 (5.52%) adverse events whereas the MTX group reported 15 (8.47%) at 12 weeks. There were 18 (5.52%) adverse events in the CCII group and 17 (9.60%) adverse events in the MTX group at 24 weeks. There were fewer and milder side effects in the CCII group than in the MTX group (Table 5). The incidence of adverse events between the CCII group and MTX group was statistically significant (*P *< 0.05).

**Table 5:** Incidence of adverse events

Adverse events	CCII (n = 326)	MTX (n = 177)
Nausea, vomiting	6	13
Abdominal pain, epigastric discomfort	17	11
Diarrhea	0	2
Liver dysfunction	6	10
Headache, dizziness	3	5
Itching, rash	5	1
Blood in urine	2	3
Constipation	1	0
Edema	2	1
Alopecia	0	1
Decrease in white blood cell count	1	5
Decrease in platelet count	0	1
Sum of events	43	53

CCII, chicken type II collagen; MTX, methotrexate.

### Laboratory variables

In the MTX group, there was a significant increase from baseline in transaminase and a decrease in white blood cell count. There were decreases in hemoglobin, platelet count, and neutrophil count in both groups, but the differences were insignificant (Table 4). Other laboratory variables were not significantly affected in the two groups.

## Discussion

Oral tolerance has been applied to prevent and treat autoimmune disease in several animal models, including arthritis. CII is the most abundant structural protein of human cartilage. The cartilage within the joint caused mainly damage of autoimmunity in patients with RA. CII autoimmunity may be a secondary phenomenon induced following inflammation in the joints and may play a role in the persistence of the disease rather than in actual induction of arthritis [25,26]. In either case, downregulation of the immune response to CII may help prevent the resulting destructive arthritis. Oral administration of CII is an established procedure for inducing peripheral immune tolerance, which suppresses autoimmune responses in RA [27-29]. Bovine or chicken CII has been administered orally to RA patients, resulting in some clinical improvement. However, the precise mechanisms of oral tolerance are not fully known. Animal studies have revealed that the mechanisms of induction of oral tolerance include clonal deletion, suppression of the pro-inflammatory Th1 cells, and the induction of regulatory T (Treg) cells. Treg cells from Peyer's patches in the gut-associated lymphoid tissue and transforming growth factor-beta (TGF-β) are reported to mediate the induction of active suppression [8,30]. Pro-inflammatory cytokines such as interleukin (IL)-1 and tumor necrosis factor-alpha downregulate and suppressive cytokines such as TGF-β and IL-4 upregulate in oral tolerance. Treg cells, defined as a persistently CD25-expressing subset of CD4^+ ^cells, may produce anti-inflammatory cytokines such as IL-10 and TGF-β and are likely to be agents of bystander suppression [31]. In a basic study, investigators in our laboratory found that CCII plays an important role in regulating the immune balance of Th1/Th2 and Th17/Treg in rats with CIA. CCII decreases the overproduction of pro-inflammatory mediator (IL-2, IL-17) and increases the hypoproduction of anti-inflammatory mediator (IL-4, TGF-β). These results may support a mechanism of oral tolerance [32].

A key feature that may affect the induction of Treg cells and other suppressive mechanisms is the dose of antigen administered. A low dose of antigen stimulates the development of Treg cells, leading to an active immune suppression. The active mechanism appears to be a cytokine-mediated immune deviation with a predominant Th2 and Th3 response (TGF-β). In contrast, high-dose oral antigens lead to clonal deletion and anergy [33,34]. The active suppression of low-dose oral tolerance can also suppress an unrelated immune response (bystander suppression), paving the way for therapy of autoimmune diseases [35]. The results from human clinical trials suggest that a daily dose of significantly less than 1 mg is optimal. Similarly, data from CIA studies reveal an optimal dose above and below which there is little or no immune suppression. Indeed, the incorrect dose can prime the immune response and aggravate disease. The timing and frequency of administration are also vital to the level of immune tolerance induced and the control of the pathological process [36].

In a multicenter study, Barnett and colleagues [14] treated 90 RA patients with CCII for 12 weeks. Patients taking the lowest dose of CII (20 μg) had a 'significant' improvement in response rate compared with placebo-treated control patients (*P *< 0.035). Corrigall and Panayi [37] also reported a significant improvement in a group of RA patients treated with lyophilized CII. None of the patients in either study had significant side effects attributable to treatment with collagen. Similarly, Barnett and colleagues [13] previously reported that 8 of 10 juvenile RA patients had decreased numbers of swollen and tender joints after 3 months of treatment with CCII. Sieper and colleagues [38] administered bovine CII orally to patients with early RA for 12 weeks using doses of either 1 mg/day or 10 mg/day. More patients in the CII-treated groups met the ACR-20 and ACR-50 improvement criteria than did patients in the placebo group [23].

On the basis of the above, CCII was developed as a novel drug of immunologic tolerance. The present study was undertaken to further evaluate whether oral administration of CCII is safe and effective in patients with RA. In this phase III trial, CCII at 0.1 mg daily and MTX at 10 mg once weekly effectively alleviated signs and symptoms of active RA. However, the efficacy of CCII did not exceed that of MTX. The incidence of treatment-related adverse events was significantly different between the CCII group and the MTX group. According to the former study, CCII led to few adverse events in patients with RA [27]. In this study, treatment was carried out in combination with diclofenac sodium, an NSAID, which can frequently cause gastrointestinal complaints. The use of diclofenac sodium can relieve pain. Nevertheless, its mechanism of action and toxicity can overlap with those of the trial drugs. The efficacy of CCII will be affected since diclofenac sodium can damage the alimentary system [39].

Above all, treatment of autoimmune diseases by induction of oral tolerance is attractive because of the few side effects and easy clinical implementation of this approach. The MTX-controlled, multicenter, 24-week trial in RA patients confirms that treatment with oral CII leads to improvement in arthritis and no significant side effects. These results are encouraging and imply that RA can be effectively treated with oral CII and partly supported the mechanism of oral tolerance. These data will provide a basis for more effective application of oral tolerance induction in RA patients. However, to clarify further the potential role and effectiveness of CCII as a toleragen in RA, ongoing studies and future work should clarify the autoimmune response to collagen in the pathogenesis of RA, and long-term observations in large numbers of patients need to confirm the efficacy of CCII and determine the optimal doses of orally administered CCII and which patients with autoimmune diseases will profit most from it.

## Conclusions

In summary, the present study provides evidence in support of the potential efficacy and safety of CCII in patients with RA. CCII significantly improved joint function and exhibited better therapeutic efficacy and is safe for human consumption, even in the long term. This study provides important information about the efficacy and safety of CCII in the treatment of RA, and this information may be useful in promoting CCII as a promising alternative therapeutic strategy that may be used as a nutritional supplement against RA.

## Abbreviations

ACR: American College of Rheumatology; ACR-20: American College of Rheumatology 20% improvement criteria; ACR-50: American College of Rheumatology 50% improvement criteria; CCII: chicken type II collagen; CIA: collagen-induced arthritis; CII: type II collagen; CRP: C-reactive protein; DAS28: disease activity score using 28 joint counts; ESR: erythrocyte sedimentation rate; HAQ: health assessment questionnaire; IL: interleukin; ITT: intention-to-treat; MTX: methotrexate; NSAID: non-steroidal anti-inflammatory drug; RA: rheumatoid arthritis; RF: rheumatoid factor; TGF-β: transforming growth factor-beta; Treg: regulatory T; VAS: visual analogue scale.

## Competing interests

The authors declare that they have no competing interests.

## Authors' contributions

WW contributed to the design of the project, served as the study coordinator, and helped to review the manuscript. LLZ contributed to the design of the project and was primarily responsible for writing the manuscript. JHX, CDB, LQN, XFL, YQW, LYS, RHZ, and BLS contributed to patient recruitment and management and to data collection. SQX, SL, WZ, JS, HXL, and RCW worked with patients to obtain informed consent, conducted clinical evaluations, took samples, and evaluated the therapeutic response to CCII. FX contributed to clinical data analysis. All authors read and approved the final manuscript.
